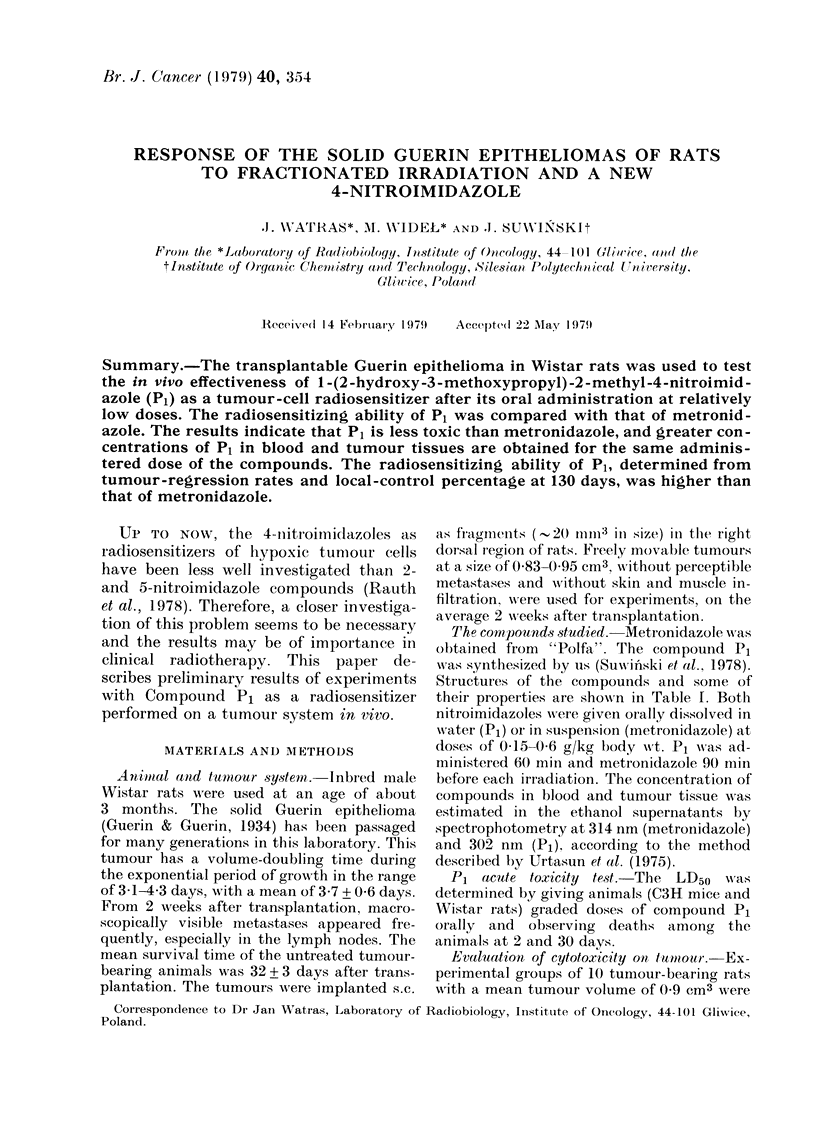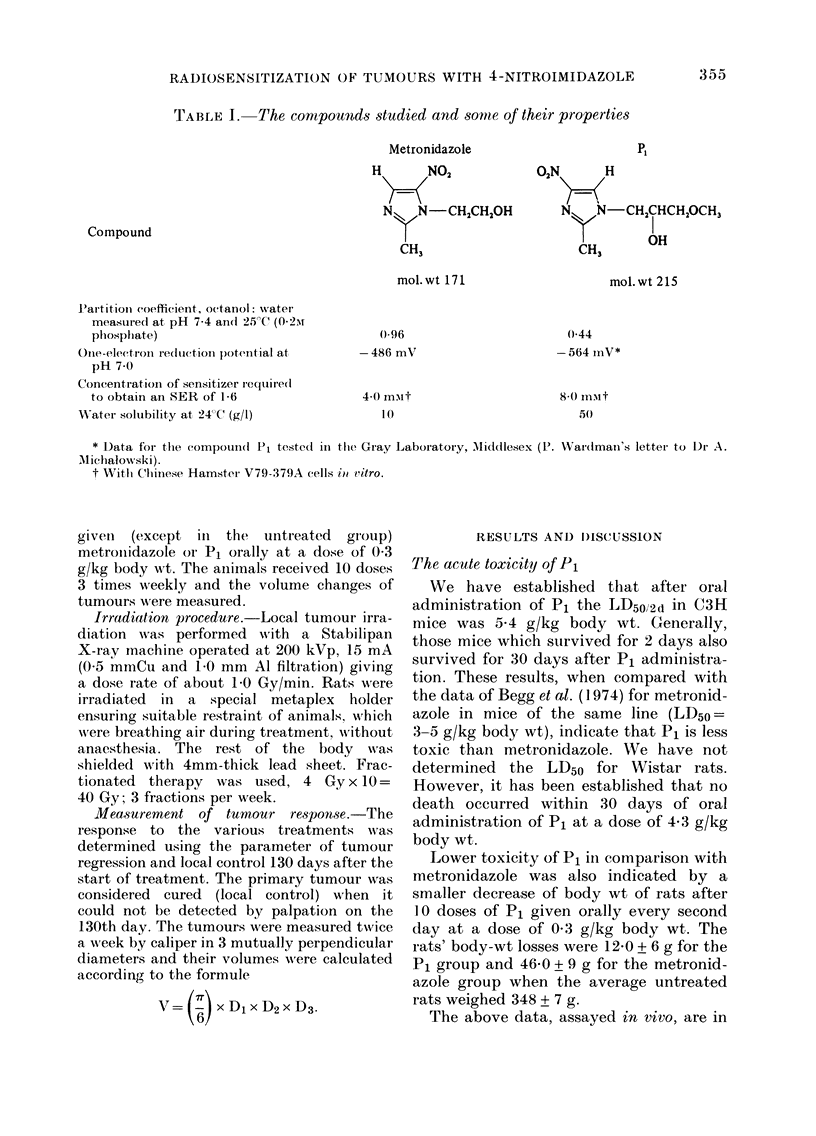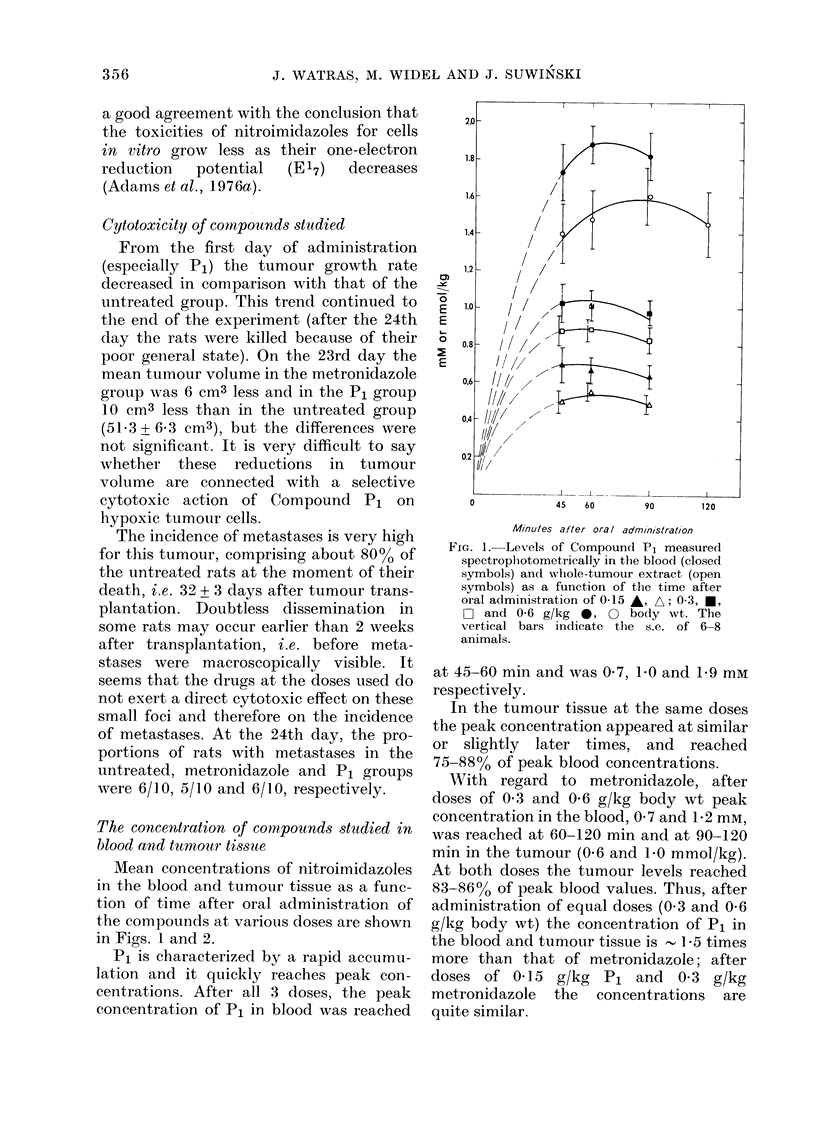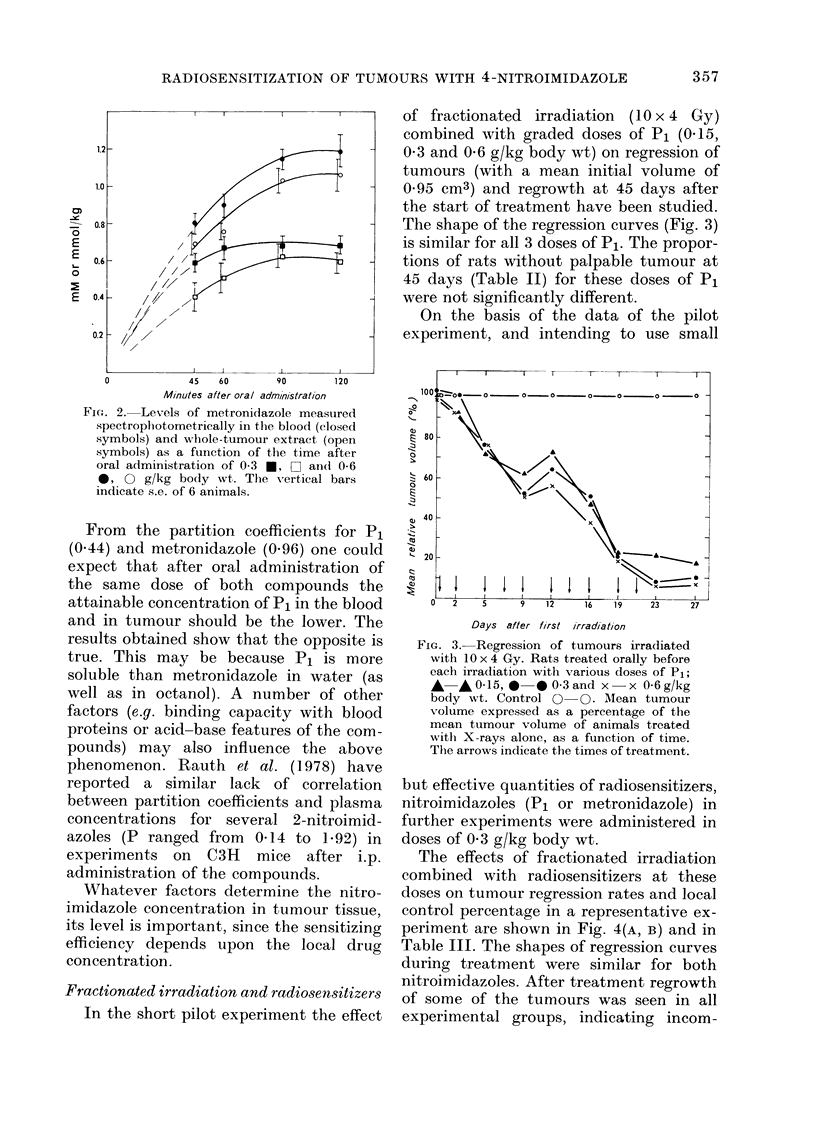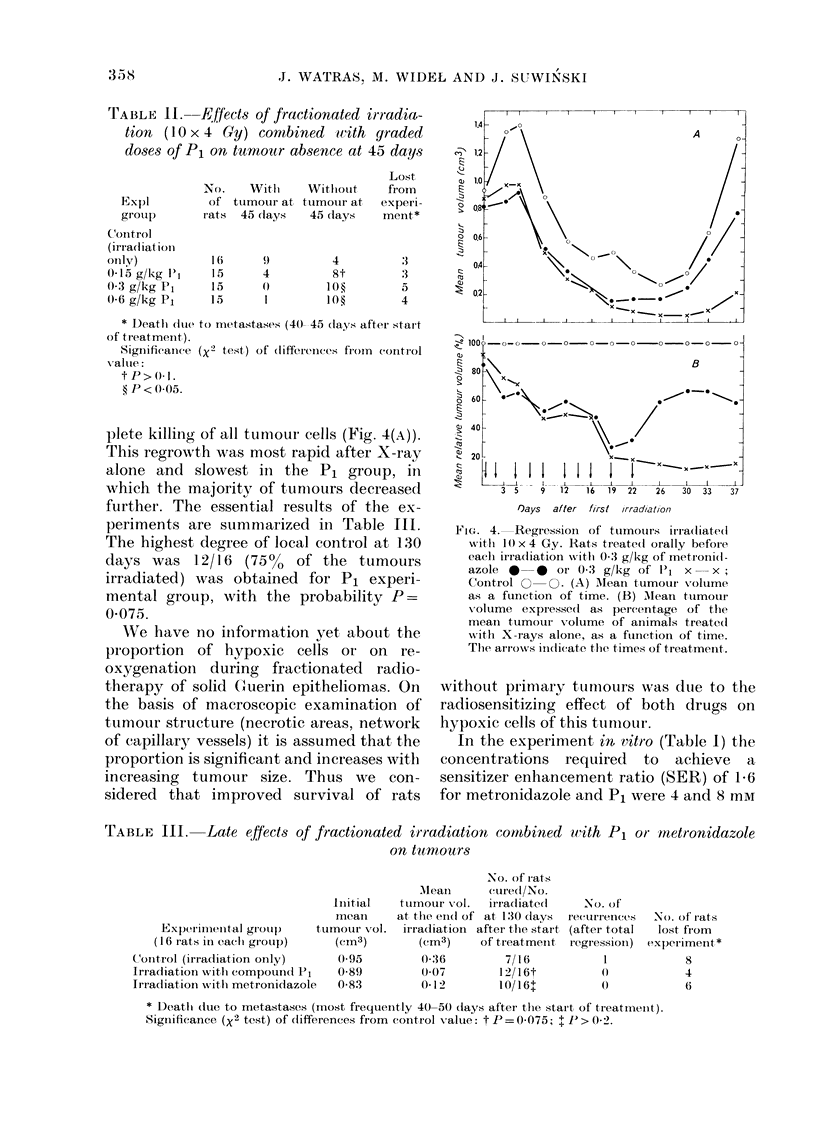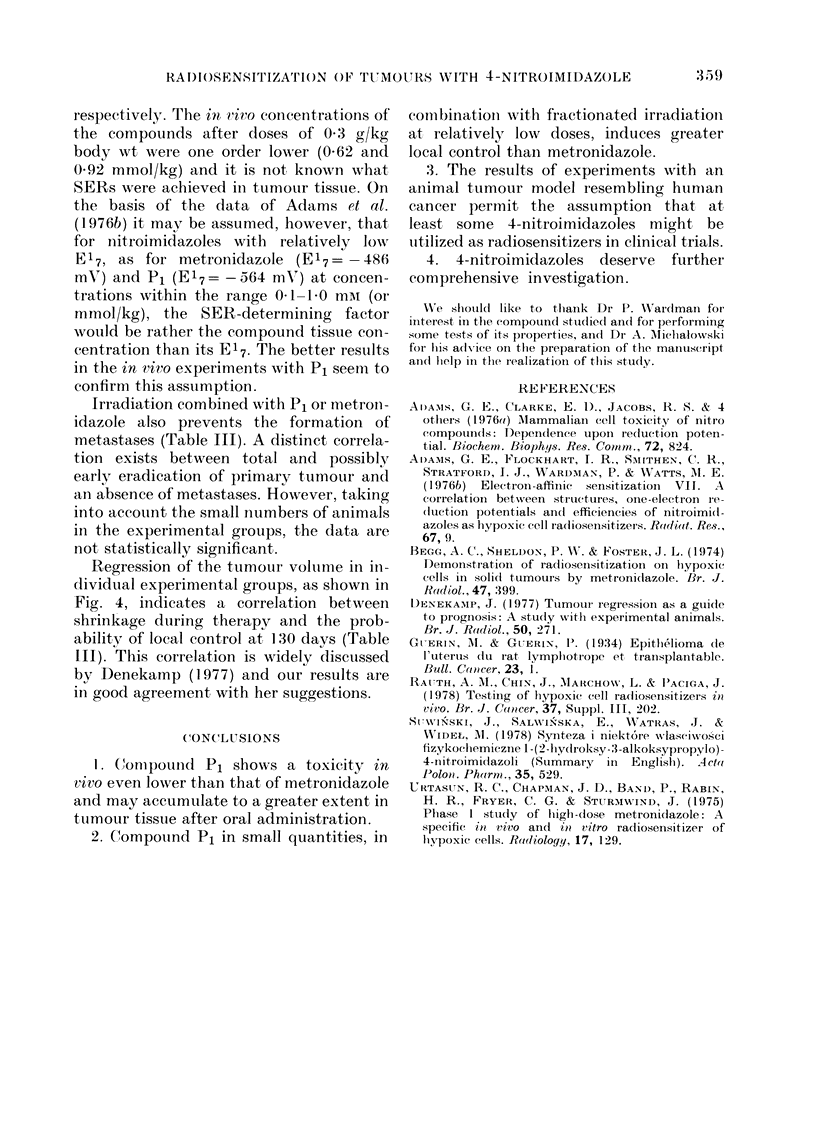# Response of the solid Guerin epitheliomas of rats to fractionated irradiation and a new 4-nitroimidazole.

**DOI:** 10.1038/bjc.1979.188

**Published:** 1979-09

**Authors:** J. Watras, M. Wideł, J. Suwiński

## Abstract

The transplantable Guerin epithelioma in Wistar rats was used to test the in vivo effectiveness of 1-(2-hydroxy-3-methoxypropyl)-2-methyl-4-nitroimidazole (P1) as a tumour-cell radiosensitizer after its oral administration at relatively low doses. The radiosensitizing ability of P1 was compared with that of metronidazole. The results indicate that P1 is less toxic than metronidazole, and greater concentrations of P1 in blood and tumour tissues are obtained for the same administered dose of the compounds. The radiosensitizing ability of P1, determined from tumour-regression rates and local-control percentage at 130 days, was higher than that of metronidazole.


*Br. J. Cancer* (1979) **40**, 354

### RESPONSE OF THE SOLID GUERIN EPITHELIOMAS OF RATS TO FRACTIONATED IRRADIATION AND A NEW 4-NITROIMIDAZOLE

J. WATRAS*, M. WIDEŁ* AND J. SUWIŃSKI†

*From the *Laboratory of Radiobiology, Institute of Oncology, 44–101 Gliwice, and the †Institute of Organic Chemistry and Technology, Silesian Polytechnical University, Gliwice, Poland*

Received 14 February 1979 Accepted 22 May 1979

**Summary.—The transplantable Guerin epithelioma in Wistar rats was used to test the *in vivo* effectiveness of 1-(2-hydroxy-3-methoxypropyl)-2-methyl-4-nitroimidazole ($P_1$) as a tumour-cell radiosensitizer after its oral administration at relatively low doses. The radiosensitizing ability of $P_1$ was compared with that of metronidazole. The results indicate that $P_1$ is less toxic than metronidazole, and greater concentrations of $P_1$ in blood and tumour tissues are obtained for the same administered dose of the compounds. The radiosensitizing ability of $P_1$, determined from tumour-regression rates and local-control percentage at 130 days, was higher than that of metronidazole.**

UP TO NOW, the 4-nitroimidazoles as radiosensitizers of hypoxic tumour cells have been less well investigated than 2- and 5-nitroimidazole compounds (Rauth *et al.*, 1978). Therefore, a closer investigation of this problem seems to be necessary and the results may be of importance in clinical radiotherapy. This paper describes preliminary results of experiments with Compound $P_1$ as a radiosensitizer performed on a tumour system *in vivo.*

### MATERIALS AND METHODS

*Animal and tumour system.*—Inbred male Wistar rats were used at an age of about 3 months. The solid Guerin epithelioma (Guerin & Guerin, 1934) has been passaged for many generations in this laboratory. This tumour has a volume-doubling time during the exponential period of growth in the range of 3·1–4·3 days, with a mean of 3·7 ± 0·6 days. From 2 weeks after transplantation, macroscopically visible metastases appeared frequently, especially in the lymph nodes. The mean survival time of the untreated tumour-bearing animals was 32 ± 3 days after transplantation. The tumours were implanted s.c. as fragments (~20 mm³ in size) in the right dorsal region of rats. Freely movable tumours at a size of 0·83–0·95 cm³, without perceptible metastases and without skin and muscle infiltration, were used for experiments, on the average 2 weeks after transplantation.

*The compounds studied.*—Metronidazole was obtained from "Polfa". The compound $P_1$ was synthesized by us (Suwiński *et al.*, 1978). Structures of the compounds and some of their properties are shown in Table I. Both nitroimidazoles were given orally dissolved in water ($P_1$) or in suspension (metronidazole) at doses of 0·15–0·6 g/kg body wt. $P_1$ was administered 60 min and metronidazole 90 min before each irradiation. The concentration of compounds in blood and tumour tissue was estimated in the ethanol supernatants by spectrophotometry at 314 nm (metronidazole) and 302 nm ($P_1$), according to the method described by Urtasun *et al.* (1975).

*$P_1$ acute toxicity test.*—The $LD_{50}$ was determined by giving animals (C3H mice and Wistar rats) graded doses of compound $P_1$ orally and observing deaths among the animals at 2 and 30 days.

*Evaluation of cytotoxicity on tumour.*—Experimental groups of 10 tumour-bearing rats with a mean tumour volume of 0·9 cm³ were

Correspondence to Dr Jan Watras, Laboratory of Radiobiology, Institute of Oncology, 44-101 Gliwice, Poland.

TABLE I.—*The compounds studied and some of their properties*

| Compound | Metronidazole  mol. wt 171 | $P_1$  mol. wt 215 |
|---|---|---|
| Partition coefficient, octanol: water measured at pH 7·4 and 25°C (0·2M phosphate) | 0·96 | 0·44 |
| One-electron reduction potential at pH 7·0 | −486 mV | −564 mV* |
| Concentration of sensitizer required to obtain an SER of 1·6 | 4·0 mM† | 8·0 mM† |
| Water solubility at 24°C (g/l) | 10 | 50 |

* Data for the compound $P_1$ tested in the Gray Laboratory, Middlesex (P. Wardman's letter to Dr A. Michałowski).

† With Chinese Hamster V79-379A cells *in vitro*.

given (except in the untreated group) metronidazole or $P_1$ orally at a dose of 0·3 g/kg body wt. The animals received 10 doses 3 times weekly and the volume changes of tumours were measured.

*Irradiation procedure.*—Local tumour irradiation was performed with a Stabilipan X-ray machine operated at 200 kVp, 15 mA (0·5 mmCu and 1·0 mm Al filtration) giving a dose rate of about 1·0 Gy/min. Rats were irradiated in a special metaplex holder ensuring suitable restraint of animals, which were breathing air during treatment, without anaesthesia. The rest of the body was shielded with 4mm-thick lead sheet. Fractionated therapy was used, 4 Gy × 10 = 40 Gy; 3 fractions per week.

*Measurement of tumour response.*—The response to the various treatments was determined using the parameter of tumour regression and local control 130 days after the start of treatment. The primary tumour was considered cured (local control) when it could not be detected by palpation on the 130th day. The tumours were measured twice a week by caliper in 3 mutually perpendicular diameters and their volumes were calculated according to the formule

$$V = \left(\frac{\pi}{6}\right) \times D_1 \times D_2 \times D_3.$$

### RESULTS AND DISCUSSION

### *The acute toxicity of $P_1$*

We have established that after oral administration of $P_1$ the $LD_{50/2d}$ in C3H mice was 5·4 g/kg body wt. Generally, those mice which survived for 2 days also survived for 30 days after $P_1$ administration. These results, when compared with the data of Begg *et al.* (1974) for metronidazole in mice of the same line ($LD_{50}$ = 3–5 g/kg body wt), indicate that $P_1$ is less toxic than metronidazole. We have not determined the $LD_{50}$ for Wistar rats. However, it has been established that no death occurred within 30 days of oral administration of $P_1$ at a dose of 4·3 g/kg body wt.

Lower toxicity of $P_1$ in comparison with metronidazole was also indicated by a smaller decrease of body wt of rats after 10 doses of $P_1$ given orally every second day at a dose of 0·3 g/kg body wt. The rats' body-wt losses were 12·0 ± 6 g for the $P_1$ group and 46·0 ± 9 g for the metronidazole group when the average untreated rats weighed 348 ± 7 g.

The above data, assayed *in vivo*, are in a good agreement with the conclusion that the toxicities of nitroimidazoles for cells *in vitro* grow less as their one-electron reduction potential ($E^1{}_7$) decreases (Adams *et al.*, 1976*a*).

*Cytotoxicity of compounds studied*

From the first day of administration (especially $P_1$) the tumour growth rate decreased in comparison with that of the untreated group. This trend continued to the end of the experiment (after the 24th day the rats were killed because of their poor general state). On the 23rd day the mean tumour volume in the metronidazole group was 6 $cm^3$ less and in the $P_1$ group 10 $cm^3$ less than in the untreated group ($51 \cdot 3 \pm 6 \cdot 3$ $cm^3$), but the differences were not significant. It is very difficult to say whether these reductions in tumour volume are connected with a selective cytotoxic action of Compound $P_1$ on hypoxic tumour cells.

The incidence of metastases is very high for this tumour, comprising about 80% of the untreated rats at the moment of their death, *i.e.* $32 \pm 3$ days after tumour transplantation. Doubtless dissemination in some rats may occur earlier than 2 weeks after transplantation, *i.e.* before metastases were macroscopically visible. It seems that the drugs at the doses used do not exert a direct cytotoxic effect on these small foci and therefore on the incidence of metastases. At the 24th day, the proportions of rats with metastases in the untreated, metronidazole and $P_1$ groups were 6/10, 5/10 and 6/10, respectively.

*The concentration of compounds studied in blood and tumour tissue*

Mean concentrations of nitroimidazoles in the blood and tumour tissue as a function of time after oral administration of the compounds at various doses are shown in Figs. 1 and 2.

$P_1$ is characterized by a rapid accumulation and it quickly reaches peak concentrations. After all 3 doses, the peak concentration of $P_1$ in blood was reached

FIG. 1.—Levels of Compound $P_1$ measured spectrophotometrically in the blood (closed symbols) and whole-tumour extract (open symbols) as a function of the time after oral administration of 0·15 ▲, △; 0·3, ■, □ and 0·6 g/kg ●, ○ body wt. The vertical bars indicate the s.e. of 6–8 animals.

at 45–60 min and was 0·7, 1·0 and 1·9 mM respectively.

In the tumour tissue at the same doses the peak concentration appeared at similar or slightly later times, and reached 75–88% of peak blood concentrations.

With regard to metronidazole, after doses of 0·3 and 0·6 g/kg body wt peak concentration in the blood, 0·7 and 1·2 mM, was reached at 60–120 min and at 90–120 min in the tumour (0·6 and 1·0 mmol/kg). At both doses the tumour levels reached 83–86% of peak blood values. Thus, after administration of equal doses (0·3 and 0·6 g/kg body wt) the concentration of $P_1$ in the blood and tumour tissue is ~ 1·5 times more than that of metronidazole; after doses of 0·15 g/kg $P_1$ and 0·3 g/kg metronidazole the concentrations are quite similar.

FIG. 2.—Levels of metronidazole measured spectrophotometrically in the blood (closed symbols) and whole-tumour extract (open symbols) as a function of the time after oral administration of 0·3 ■, □ and 0·6 ●, ○ g/kg body wt. The vertical bars indicate s.e. of 6 animals.

From the partition coefficients for $P_1$ (0·44) and metronidazole (0·96) one could expect that after oral administration of the same dose of both compounds the attainable concentration of $P_1$ in the blood and in tumour should be the lower. The results obtained show that the opposite is true. This may be because $P_1$ is more soluble than metronidazole in water (as well as in octanol). A number of other factors (*e.g.* binding capacity with blood proteins or acid–base features of the compounds) may also influence the above phenomenon. Rauth *et al.* (1978) have reported a similar lack of correlation between partition coefficients and plasma concentrations for several 2-nitroimidazoles (P ranged from 0·14 to 1·92) in experiments on C3H mice after i.p. administration of the compounds.

Whatever factors determine the nitroimidazole concentration in tumour tissue, its level is important, since the sensitizing efficiency depends upon the local drug concentration.

*Fractionated irradiation and radiosensitizers*

In the short pilot experiment the effect of fractionated irradiation (10×4 Gy) combined with graded doses of $P_1$ (0·15, 0·3 and 0·6 g/kg body wt) on regression of tumours (with a mean initial volume of 0·95 $cm^3$) and regrowth at 45 days after the start of treatment have been studied. The shape of the regression curves (Fig. 3) is similar for all 3 doses of $P_1$. The proportions of rats without palpable tumour at 45 days (Table II) for these doses of $P_1$ were not significantly different.

On the basis of the data of the pilot experiment, and intending to use small

FIG. 3.—Regression of tumours irradiated with 10×4 Gy. Rats treated orally before each irradiation with various doses of $P_1$; ▲—▲ 0·15, ●—● 0·3 and ×—× 0·6 g/kg body wt. Control ○—○. Mean tumour volume expressed as a percentage of the mean tumour volume of animals treated with X-rays alone, as a function of time. The arrows indicate the times of treatment.

but effective quantities of radiosensitizers, nitroimidazoles ($P_1$ or metronidazole) in further experiments were administered in doses of 0·3 g/kg body wt.

The effects of fractionated irradiation combined with radiosensitizers at these doses on tumour regression rates and local control percentage in a representative experiment are shown in Fig. 4(A, B) and in Table III. The shapes of regression curves during treatment were similar for both nitroimidazoles. After treatment regrowth of some of the tumours was seen in all experimental groups, indicating incom-

TABLE II.—*Effects of fractionated irradiation (10 × 4 Gy) combined with graded doses of $P_1$ on tumour absence at 45 days*

| Expl group | No. of rats | With tumour at 45 days | Without tumour at 45 days | Lost from experiment* |
|---|---|---|---|---|
| Control (irradiation only) | 16 | 9 | 4 | 3 |
| 0·15 g/kg $P_1$ | 15 | 4 | 8† | 3 |
| 0·3 g/kg $P_1$ | 15 | 0 | 10§ | 5 |
| 0·6 g/kg $P_1$ | 15 | 1 | 10§ | 4 |

* Death due to metastases (40–45 days after start of treatment).

Significance ($\chi^2$ test) of differences from control value:

§ $P < 0·05$.

FIG. 4.—Regression of tumours irradiated with 10 × 4 Gy. Rats treated orally before each irradiation with 0·3 g/kg of metronidazole ●—● or 0·3 g/kg of $P_1$ ×—×; Control ○—○. (A) Mean tumour volume as a function of time. (B) Mean tumour volume expressed as percentage of the mean tumour volume of animals treated with X-rays alone, as a function of time. The arrows indicate the times of treatment.

plete killing of all tumour cells (Fig. 4(A)). This regrowth was most rapid after X-ray alone and slowest in the $P_1$ group, in which the majority of tumours decreased further. The essential results of the experiments are summarized in Table III. The highest degree of local control at 130 days was 12/16 (75% of the tumours irradiated) was obtained for $P_1$ experimental group, with the probability $P =$ 0·075.

We have no information yet about the proportion of hypoxic cells or on reoxygenation during fractionated radiotherapy of solid Guerin epitheliomas. On the basis of macroscopic examination of tumour structure (necrotic areas, network of capillary vessels) it is assumed that the proportion is significant and increases with increasing tumour size. Thus we considered that improved survival of rats without primary tumours was due to the radiosensitizing effect of both drugs on hypoxic cells of this tumour.

In the experiment *in vitro* (Table I) the concentrations required to achieve a sensitizer enhancement ratio (SER) of 1·6 for metronidazole and $P_1$ were 4 and 8 mM

TABLE III.—*Late effects of fractionated irradiation combined with $P_1$ or metronidazole on tumours*

| Experimental group (16 rats in each group) | Initial mean tumour vol. (cm³) | Mean tumour vol. at the end of irradiation (cm³) | No. of rats cured/No. irradiated at 130 days after the start of treatment | No. of recurrences (after total regression) | No. of rats lost from experiment* |
|---|---|---|---|---|---|
| Control (irradiation only) | 0·95 | 0·36 | 7/16 | 1 | 8 |
| Irradiation with compound $P_1$ | 0·89 | 0·07 | 12/16† | 0 | 4 |
| Irradiation with metronidazole | 0·83 | 0·12 | 10/16‡ | 0 | 6 |

* Death due to metastases (most frequently 40–50 days after the start of treatment).

Significance ($\chi^2$ test) of differences from control value: † $P = 0·075$; ‡ $P > 0·2$.

respectively. The *in vivo* concentrations of the compounds after doses of 0·3 g/kg body wt were one order lower (0·62 and 0·92 mmol/kg) and it is not known what SERs were achieved in tumour tissue. On the basis of the data of Adams *et al.* (1976*b*) it may be assumed, however, that for nitroimidazoles with relatively low $E^1_7$, as for metronidazole ($E^1_7 = -486$ mV) and $P_1$ ($E^1_7 = -564$ mV) at concentrations within the range 0·1–1·0 mM (or mmol/kg), the SER-determining factor would be rather the compound tissue concentration than its $E^1_7$. The better results in the *in vivo* experiments with $P_1$ seem to confirm this assumption.

Irradiation combined with $P_1$ or metronidazole also prevents the formation of metastases (Table III). A distinct correlation exists between total and possibly early eradication of primary tumour and an absence of metastases. However, taking into account the small numbers of animals in the experimental groups, the data are not statistically significant.

Regression of the tumour volume in individual experimental groups, as shown in Fig. 4, indicates a correlation between shrinkage during therapy and the probability of local control at 130 days (Table III). This correlation is widely discussed by Denekamp (1977) and our results are in good agreement with her suggestions.

### CONCLUSIONS

1. Compound $P_1$ shows a toxicity *in vivo* even lower than that of metronidazole and may accumulate to a greater extent in tumour tissue after oral administration.

2. Compound $P_1$ in small quantities, in combination with fractionated irradiation at relatively low doses, induces greater local control than metronidazole.

3. The results of experiments with an animal tumour model resembling human cancer permit the assumption that at least some 4-nitroimidazoles might be utilized as radiosensitizers in clinical trials.

4. 4-nitroimidazoles deserve further comprehensive investigation.

We should like to thank Dr P. Wardman for interest in the compound studied and for performing some tests of its properties, and Dr A. Michałowski for his advice on the preparation of the manuscript and help in the realization of this study.

### REFERENCES

Adams, G. E., Clarke, E. D., Jacobs, R. S. & 4 others (1976*a*) Mammalian cell toxicity of nitro compounds: Dependence upon reduction potential. *Biochem. Biophys. Res. Comm.*, **72**, 824.

Adams, G. E., Flockhart, I. R., Smithen, C. R., Stratford, I. J., Wardman, P. & Watts, M. E. (1976*b*) Electron-affinic sensitization VII. A correlation between structures, one-electron reduction potentials and efficiencies of nitroimidazoles as hypoxic cell radiosensitizers. *Radiat. Res.*, **67**, 9.

Begg, A. C., Sheldon, P. W. & Foster, J. L. (1974) Demonstration of radiosensitization on hypoxic cells in solid tumours by metronidazole. *Br. J. Radiol.*, **47**, 399.

Denekamp, J. (1977) Tumour regression as a guide to prognosis: A study with experimental animals. *Br. J. Radiol.*, **50**, 271.

Guerin, M. & Guerin, P. (1934) Epithélioma de l'uterus du rat lymphotrope et transplantable. *Bull. Cancer*, **23**, 1.

Rauth, A. M., Chin, J., Marchow, L. & Paciga, J. (1978) Testing of hypoxic cell radiosensitizers *in vivo*. *Br. J. Cancer*, **37**, Suppl. III, 202.

Suwiński, J., Salwińska, E., Watras, J. & Wideł, M. (1978) Synteza i niektóre właściwości fizykochemiczne 1-(2-hydroksy-3-alkoksypropylo)-4-nitroimidazoli (Summary in English). *Acta Polon. Pharm.*, **35**, 529.

Urtasun, R. C., Chapman, J. D., Band, P., Rabin, H. R., Fryer, C. G. & Sturmwind, J. (1975) Phase I study of high-dose metronidazole: A specific *in vivo* and *in vitro* radiosensitizer of hypoxic cells. *Radiology*, **17**, 129.